# Musculoskeletal model predicted paraspinal loading may quick estimate the effect of exercise on spine BMD

**DOI:** 10.3389/fbioe.2024.1464067

**Published:** 2024-12-23

**Authors:** Shizhong Liu, Xiaoyu Xia, Yinxia Nie, Mengen Huang, Lin Meng, Juan Du

**Affiliations:** ^1^ Academy of Medical Engineering and Translational Medicine, Tianjin University, Tianjin, China; ^2^ Rehabilitation Department, Tianjin Medical University General Hospital, Tianjin, China; ^3^ College of Sports Health, Tianjin University of Sport, Tianjin, China

**Keywords:** musculoskeletal model, exercise, spine biomechanics, bone remodelling, osteoporosis

## Abstract

**Purpose:**

Spine is the most commonly found fracture site due to osteoporosis. Combined exercise including high-impact and resistance exercise shows the potential to improve bone mineral density (BMD) in the spine. However, the mechanical loading introduced by exercise, which is the mechanism of BMD changes, has not been investigated. The purpose of this study is to provide a new insight to investigate the mechanical stimuli of exercise induced bone remodelling.

**Methods:**

Ten postmenopausal women with osteopenia who finalized a 6-month combined exercise of high-impact and resistance intervention were included. The changes in BMD were analyzed based on QCT images obtained from pre and post intervention. A modified full-body musculoskeletal model was built to estimate paravertebral muscle force (MF) and intervertebral compression force (ICF) during daily activities (walking and heel drop) and combined exercise including high-impact (jumping) and resistance exercise (pulling elastic bands).

**Results:**

The paravertebral MF and ICF during jumping and pulling elastic bands exercise were all significantly greater than walking and heel drop exercise with up to 1.22–8.18 times. Spine BMD remained at the same level with no significant decline observed, especially at L1 (pre 247.95 ± 26.77 mg/cm^3^ and post 245.49 ± 22.04 mg/cm^3^). Comparing with daily activities, significant correlations were observed between the changes of BMD and the sum of spinal loadings generated by combined exercise at both global and segmental level (r = 0.687, *p* < 0.05).

**Conclusion:**

It has been proved that paravertebral muscle forces and intervertebral compression forces generated by the combined exercise, rather than daily exercise, were the main reasons for the improvement of spine BMD. This study contributes into the understanding of exercise induced spine adaptation as well as a potential in fast prediction to evaluate the effect of physical exercise therapy.

## 1 Introduction

Osteoporosis (OP) is the most common chronic metabolic bone disease characterized by a systemic impairment of bone mineral density (BMD) and microarchitecture (Lin and Lane, 2004). It can lead to pain, kyphosis, and an increase in the risk of fragility fractures (Rachner et al., 2011). One-third of women over the age of 50 years experienced osteoporotic fractures in their lifetime (Sözen et al., 2017), where the spine is the most commonly found fracture (Kistler-Fischbacher et al., 2021). Previous studies have shown that exercise can effectively increase BMD and slow down bone loss (Zhang et al., 2022; Du et al., 2021a). However, the relationships of exercise induced BMD changes and spinal loading is still under investigation.

Most of the previous exercise interventions focused on observing the effect of different exercise programs on BMD changes. A study designed a 1-year brisk walking training program and found that it did not significantly improve spine BMD (Brooke-Wavell et al., 1997). Another 8-month follow-up experiment included an exercise group (EG) performing heel drops, muscle endurance, and balance exercises, while the control group (CG) maintained their usual lifestyle. The results indicated that this exercise program did not significantly improve spine BMD (Marques et al., 2011). A meta-analysis synthesized current evidence from 24 clinical trials. It was found that combined resistance exercise protocols appear effective in spine BMD in postmenopausal women, whereas resistance-alone protocols only produced a nonsignificant positive effect (Zhao et al., 2015). Dual-energy X-ray absorptiometry (DXA) was used to observe the changes in BMD, however, it can only provide two-dimensional images and average BMD of the global spine. Quantitative computed tomography (QCT) was superior to DXA for certain indications because it is a better morphological assessment of the spine (Link and Lang, 2014; Du et al., 2019). Volumetric BMD measured by QCT is less susceptible to interference from degenerative changes in the measurement site and soft tissue calcification, resulting in more accurate BMD measurements. Previous studies showed that QCT can also measure three-dimensional structural information of bone (Gerety et al., 2017). Therefore, QCT serves as an essential method to address the limitations of DXA in assessing BMD changes (Allison et al., 2015). However, most studies based on QCT observed the changes in BMD phenomenally through exercise programs over 6 months. The change in BMD is highly sensitive to the exercise programs (Engelke et al., 2006; Fountoulis et al., 2016). This sensitivity may be attributed to significant differences in spine loading generated by different exercise programs. Currently, no studies investigated the mechanical loading of different exercise programs, which is the fundamental stimuli of BMD changes.

According to Wolff’s law, mechanical stimulus cause changes in bone mass, shape, and microstructure (Mellon and Tanner, 2012). Therefore, the mechanical stimuli generated by exercise are the fundamental reason for the improvement in spine BMD. Ground reaction force (GRF) was analyzed to represent the mechanical loading caused by exercises. However, GRF may not accurately reflect joint force information in regions like the spine. For example, a study evaluated the maximum voluntary forefoot GRF during multiple one-legged hopping to determine the correlation with tibial volumetric bone mineral content (vBMC) (Anliker et al., 2011). The results only found a significant correlation between GRF and vBMC at the tibia, but no statistically significant were found for other regions. This may be due to substantial differences in the mechanical loading experienced by joints such as the knee, hip, and spine during exercise. Therefore, to explain the various effects of different exercises on BMD, a method that can accurately calculate spinal loading needs to be developed.

Implanted sensors were used occasionally to measure biomechanics of the spine in some studies. For instance, measuring the intervertebral disc pressure (IDP) involves inserting pressure sensors into the intervertebral discs (Nachemson and Morris, 1964; Nachemson, 1965), while the forces and moments applied on the spine were measured from the sensors. However, sensors have to be invasively positioned into humans. Consequently, non-invasive, image-based modelling approaches have emerged as an alternative for predicting spine biomechanical behaviors. Musculoskeletal (MS) models provide a non-invasive method to study human activities and predict mechanical loading including intervertebral compression force (ICF) and paravertebral muscle force (MF) during exercise (Delp et al., 2007). Muscle-muscle and muscle-bone interactions were accounted for (Li et al., 2019; Abe et al., 2000). Many researchers have established and validated MS models, such as the spine (Christophy et al., 2012), and wrist (McFarland et al., 2022), to simulate various movement tasks, predict joint loads and muscle activation. A full-body MS model encompassing the lower limbs and the spine has been used to simulate a range of spinal movements during daily activities and predict joint forces during different exercises. Additionally, previous research has established an MS model incorporating a rigid pelvis, sacrum, spine, and torso (Christophy et al., 2012). This model predicted joint forces, muscle activation patterns, and forecasted MF arms during flexion-extension movements in the lower back, validating their physiological similarity. Furthermore, the full-body MS model has been used to estimate spine loads and MFs during asymmetric lifting tasks (Kim and Zhang, 2017). They identified higher peak lateral shear forces and paraspinal MFs during asymmetric lifting, potentially increasing the risk of lower back injuries. However, MS models have not been used to predict the mechanical loads on the spine in different impact (walking, heel drop, jumping) and resistance exercise programs, explaining the various effects of different exercises on spine BMD.

Therefore, in this study, we aim to investigate the mechanical loading of exercise and its relationships with BMD changes in the spine. A full-body MS model was modified which can be used to predict mechanical loading at the spine including paravertebral MF and ICF during walking, heel drop, jumping and pull elastic bands. We also reported the effect of 6-month combined exercise in healthy postmenopausal women on spine BMD. Finally, to investigate the relationships between BMD changes and spinal load caused by different exercises and insight the mechanism of exercise induced bone remodelling.

## 2 Methods

### 2.1 Participants and study design

In this study, 15 postmenopausal women with osteopenia (age 58.29 ± 7.28 years, weight 58.89 ± 7.07 kg, height 159.84 ± 6.27 cm) were recruited from a cohort of a 6-month combined exercise experiment (registered at Chinese clinical trials: ChiCTR2400081574: The Effect of High Impact and Resistance Exercise on Spine and Articular Cartilage in Chinese Post-menopausal Women). The inclusion criteria were (1) menopause time ≥1 year; (2) individuals who had osteopenia (T-score between −1.0 and −2.5 SD) (3) BMI below 30 kg/m^2^, and (4) not taking medication known to affect BMD. Concomitantly, eligible participants were invited to perform DXA and QCT scans. Eligibility was also assessed based on BMI and risk factors for osteoporosis. All subjects perform the same exercise protocol. Briefly, participants were asked to attend supervised training sessions on campus twice a week, involving activities high-impact and resistance exercises such as rope skipping with vertical landing (5 sets of 15 repetitions), resistance band exercises (4 sets of 10 repetitions), and a stretching routine. Compliance was assessed through participant diaries. Meanwhile, the participants maintained their lifestyle. A survey was used to find out their daily activities, walking and heel drop found the two of the common activities in daily life. Considering the bone remodelling cycle was approximately 4–6 months, a 6-month experimental period was chosen (Zhao et al., 2017; Du et al., 2021b). During the initial screening visit, all participants received a detailed explanation of the study’s purpose, procedures, and risks. Written informed consent was obtained from all participants prior to experiment (Approval Number: TJUE-2022-141). This study adheres to CONSORT guidelines. Of the 15 participants who participated in this study, 10 participants (age 59.56 ± 7.18 years, weight 59.83 ± 7.59 kg, height 160.78 ± 5.45 cm) completed the 6-month intervention corresponding to attrition rates of 33.3%. One participant in the, EG dropped out because of occupational changes and another because of an unrelated severe disease. Three participants in the, EG were excluded from the analysis due to poor training adherence, defined as an average of less than two exercise sessions per week throughout the study period.

**FIGURE 1:**
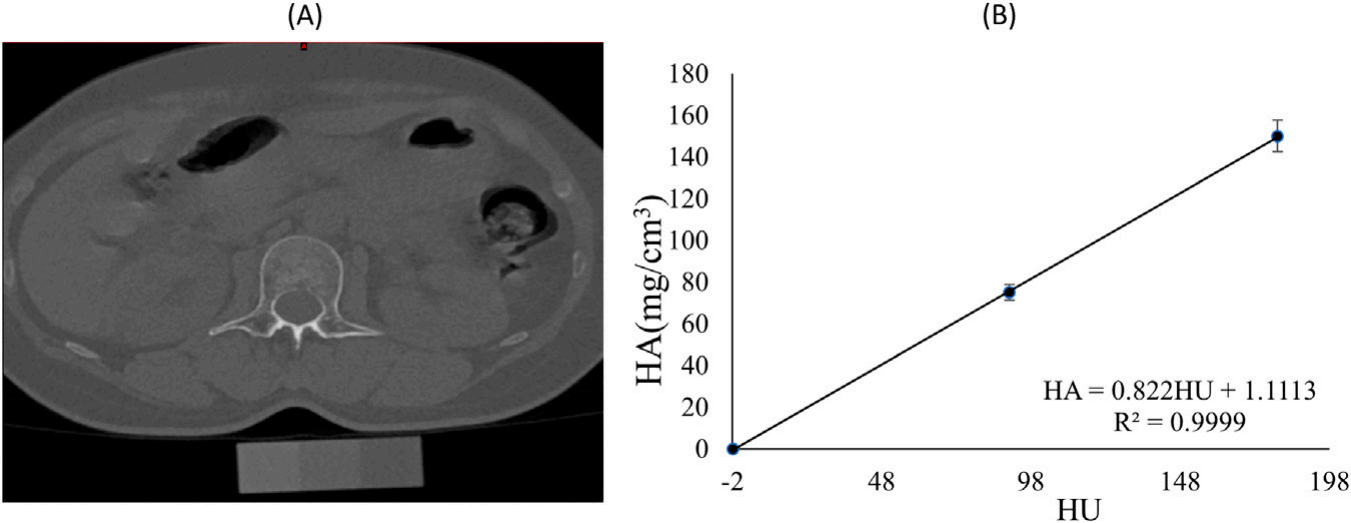
**(A)** Spine CT image and phantom of a participant; **(B)** Calibration equation for Hounsfield units (HU) and hydroxyapatite (HA) mineral density for the same participant.

QCT (Somatom Sensation 64, Siemens, Germany) images of the vertebrae T12 to S1 were obtained using the manufacturer’s standard *in vivo* protocol described (120 kV, 20 mA). Images were 5-mm section thickness, 512 × 512 planar pixel resolution and 0.488 × 0.488 mm planar pixel spacing. The QCT machine was calibrated to make sure linear attenuation of the phantom was converted to hydroxyapatite (HA) densities and calibrated on a daily basis following manufacturer’s standard method. The vertebrae L1-L5 was separated by a fixed threshold algorithm provided by the segmentation software (Materialise, Leuven, Belgium). The BMD values of entire individual lumbar vertebrae segments were analyzed. Specifically, BMD was converted according to linear regression between the three-element calibration phantom with predefined HA (0, 75, 150 mg/cm^3^) and HU values in the images (Figure 1). After 6 months, the participants were invited back for additional QCT scans and anthropometry to complete data collection.

### 2.2 The musculoskeletal model

A full-body spine (FBLS) model (Raabe and Chaudhari, 2016) was modified in Opensim4.1 (SimTK, Stanford, CA). The purpose of developing this model was to provide a comprehensive model suitable for studies involving the spine. The model contained detailed spine information, containing 21 body segments, 30 degrees of freedom, and 324 muscle-tendon actuators. To study the biomechanical effects of different exercises, this study established comprehensive MS models suitable for impact exercise (walking, heel drop, and jumping), as well as resistance exercises (pulling elastic bands).

**FIGURE 2:**
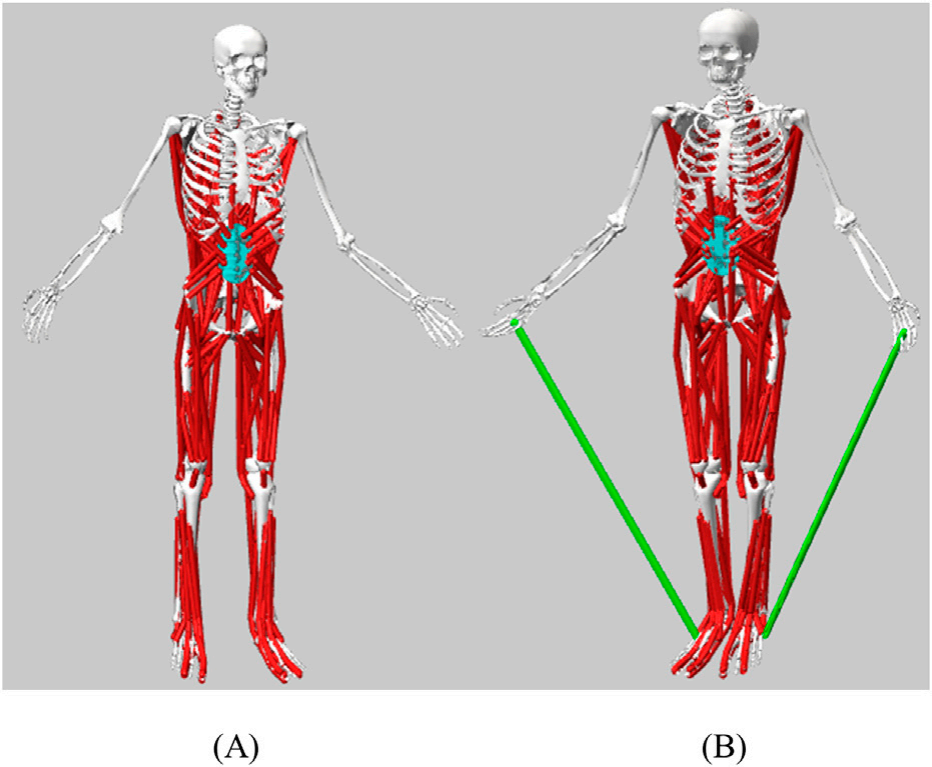
**(A)** Impact exercise MS model and **(B)** resistance exercise MS model.

Impact exercise MS model Figure 2A: The original FBLS model had no adjustments to any joint degrees of freedom. Resistance exercise MS model Figure 2B: The upper limb flexion-extension range of motion in the FBLS model was modified from −90°−90° to −90°−180° to better reflect the actual conditions of resistance exercise. Springs were added on both sides of the model, with each spring connecting the palm and heel bone on the same side to simulate elastic resistance bands. The rest length of the spring was set to 0.35 m, and the stiffness parameter was set to 28.29 N/m. These parameters of the spring were measured by tensile tests.

**FIGURE 3:**
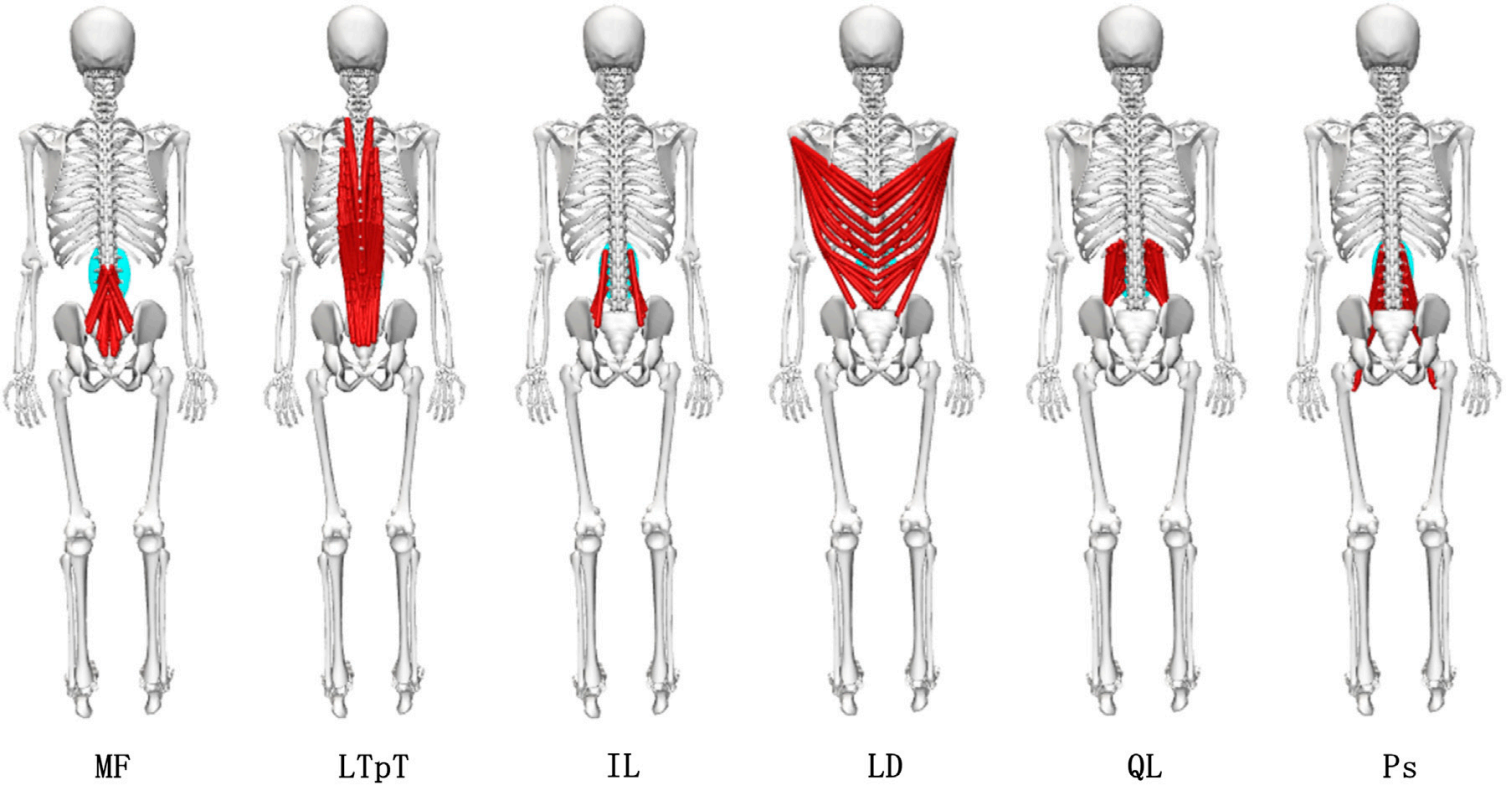
Diagram of the muscle groups of the paraspinal muscles in the musculoskeletal model.

The model simulations were conducted in Opensim4.1. Both full-body MS models needed to be scaled according to experimental data to match the geometric dimensions of the participants. Subsequently, inverse kinematics (IK), residual reduction algorithm (RRA), and static optimization (SO) were performed to estimate paraspinal MFs and muscle activations during exercises (Ueno et al., 2017; Mokhtarzadeh et al., 2013). The model calculated MFs using the algorithm that minimized the sum of squared muscle activations (Delp et al., 2007).

$$J=\sum_{m=1}^{n}{\left(a_{m}\right)}^{p}$$

where n is the number of muscles in the model; and a_m_ is the activation level of muscle m at a discrete time step. Joint Reaction (JR) analyse (Aghdam et al., 2022) is a complicated dynamic analysis process, the formula for calculating usually involves Newton-Euler equation and motion equation.

$$F_{joint}=M_{total}*a_{joint}-{\sum F}_{external}$$

where $F_{joint}$ is joint force; $M_{total}$ is the mass of body; $a_{joint}$ is joint acceleration; ${\sum F}_{external}$ is the sum of all external forces applied to the model. Joint Reaction (JR) analyze was employed to determine the ICF in the model under the combined influence of kinematics, external loads, and internal loads. The paraspinal muscles in the musculoskeletal model include the multifidus (MF), longissimus thoracis pars thoracis (LTpT), iliocostalis lumborum (IL), latissimus dorsi (LD), quadratus lumborum (QL), and psoas major (Ps). It was shown in Figure 3.

### 2.3 Data collection

Kinematic and dynamic data were collected for 6-month exercise program, which engaged in jumping and resistance (pulling elastic bands) exercise, and daily exercise, which involved walking and heel drop. Participants were instructed to perform the designated exercises. For the walking task, participants were required to move at a normal pace, alternating between left and right feet over the central position of 4 force plates. Heel drop involves lifting the heels, concentrating body weight on the toes, and then allowing the heels to descend freely. Participants were instructed to maintain an upright posture with hands at their sides throughout the entire process. The jumping activity required participants to exert maximum effort in a vertical jump, allowing free arm swinging, similar to jump rope exercises. In the resistance exercise, participants were instructed to step on an elastic band, tightly gripping both ends with their hands, and slowly raise it over their heads before lowering it.

**FIGURE 4:**
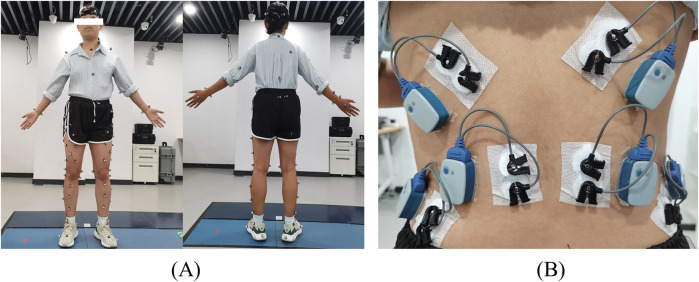
The placement of reflective markers **(A)** and the placement of electromyography sensors **(B)**.

Kinematic and dynamic data were sampled at 100 Hz using a motion capture system comprising 15 cameras from VICON (VICON motion system, Ltd., Oxford, United Kingdom) and 3 force plates (BP400600, AMTI, Watertown, United States). Reflective markers were placed on segments of the body, including the head, torso, arms, pelvis, and lower limbs, totalling 77 markers to track full-body activities. The placements of reflective markers were shown in Figure 4A.

The FBLS model was only validated for jogging; hence, further validation of the model’s accuracy was required for the 4 exercises in this study. A direct validation method of the simulation results was performed by comparing experimental electromyography (EMG) activity recorded from trunk muscles with the model-estimated muscle activations (van den Bogert et al., 2008). Participants were asked to perform the aforementioned 4 exercises, each repeated 3 times. Kinematic and dynamic data during these exercises were collected. Six electromyography electrodes (Noraxon United States, Inc.) were placed on both sides of the longissimus thoracis muscle (LTpT), latissimus dorsi (LD), and external oblique (EO) muscles, synchronously sampling EMG signals at a frequency of 2000 Hz. The placement of electromyography sensors were shown in Figure 4B. To obtain muscle activation intensity, surface EMG signals (Alemi et al., 2023) were 20–450 Hz bandpass filtered, rectified, low-pass filtered at 6 Hz, and normalized. The activations of multiple muscle bundles represented in the model, corresponding to the electrodes in the experiment, were summed and normalized. The experimental EMG activity recorded for the trunk muscles was compared to the muscle activation estimated by the model. Furthermore, the 2 curves were compared using a cross-correlation analysis (xcorr function in MATLAB, The MathWorks Inc., United States); the mean peak cross-correlation coefficient was obtained by averaging these values across 3 trials of each condition within a task.

### 2.4 Statistical analysis

The one-way analysis of variance (ANOVA) was employed to describe the statistical significance of paraspinal MF and ICF changes among walking, heel drop, and jumping, as well as resistance exercises.

**FIGURE 5:**
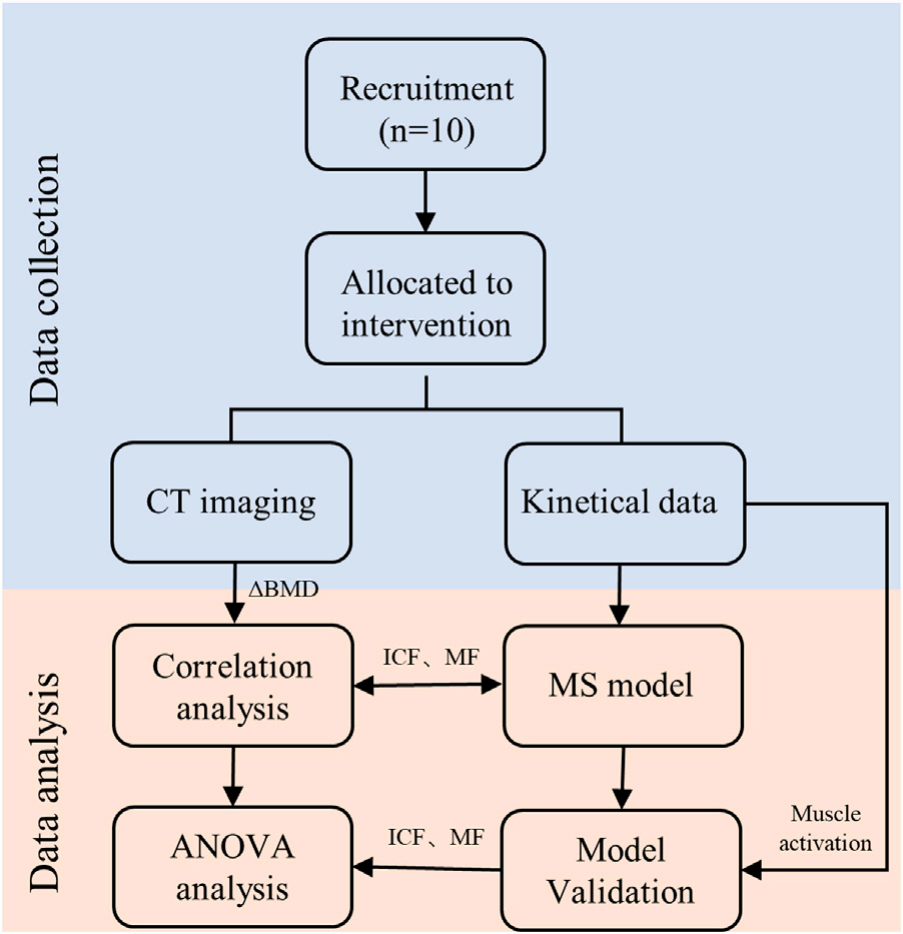
Workflow diagram of the study design.

T-test for paired samples was used for the assessment of the means obtained for BMD values from the pre and post intervention. One-way analysis of variance (ANOVA) was used for the assessment of the statistical significance of BMD among L1-L5 segments. Subsequently, the Pearson correlation test and linear regression analyzes were performed to establish a unified pattern between the paravertebral MF and ICF during combined exercises and the changes in BMD across the 5 spine segments. Figure 5 is the overall workflow diagram of the study design. All analyzes were carried out using SPSS Statistics (version 23; IBM Corp., NY, United States), *p*-value less than 0.05 was considered statistically significant.

## 3 Results

### 3.1 Changes of BMD at different vertebral segments

Spine BMD measured at pre and post intervention was shown in Table 1. The study found that after 6 months of exercise intervention, the L1-L5 segments BMD exhibited a decreasing trend, but the results of paired sample *t*-test showed that the decrease in BMD of each segment was not significant (*p* > 0.05). L1 segment BMD decreased the least (−0.82% ± 2.12%, *p* = 0.241). This was followed by L2 (−0.86% ± 2.32%, *p* = 0.26), L5 (−1.11% ± 2.43%; *p* = 0.14) and L4 (−1.43% ± 2.76%; *p* = 0.149).

**TABLE 1:** The BMD of 10 participants pre and post intervention.

BMD mg/cm^3^	Participants (n = 10)			
	Pre	Post	Δ%	*p*-Value
L1	247.95 ± 26.77	245.49 ± 22.04	−0.82 ± 2.12	0.241
L2	260.83 ± 34.16	258.10 ± 29.53	−0.86 ± 2.32	0.260
L3	265.72 ± 36.16	260.54 ± 31.81	−1.78 ± 2.67	0.085
L4	268.73 ± 32.49	264.52 ± 29.08	−1.43 ± 2.76	0.149
L5	266.49 ± 37.08	262.91 ± 31.88	−1.11 ± 2.43	0.175

Δ% indicates changes in percentage to 6 months.

It was observed that the distribution pattern of BMD in each lumbar segment pre intervention was consistent with that post intervention, showing a gradual increase in L1-L5. In addition, there was no significant difference in BMD of each lumbar segment pre and post intervention by one-way ANOVA (*p* > 0.05). All samples were tested for normality and conformed to the normal distribution.

### 3.2 MS model validation

**FIGURE 6:**
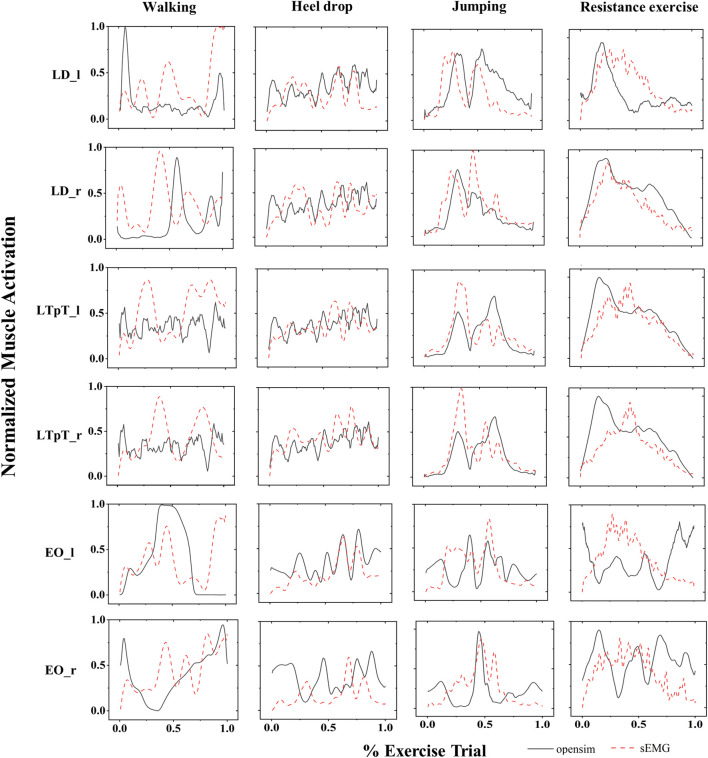
The normalized sEMG signals (red dashed lines) and the corresponding MS model simulation values (black solid lines) for the left external oblique (EO_l), right external oblique (EO_r), left pectoralis major (LTpT_l), right pectoralis major (LTpT_r), left latissimus dorsi (LD_l), and right latissimus dorsi (LD_r) are shown for the 4 types of exercises.

Figure 6 shows the average surface electromyogram signal (sEMG) signals of the bilateral back muscles collected during 3 repetitions of walking, heel drop, jumping, and resistance exercises. Additionally, the muscle activation estimated by the MS model was presented in comparison with sEMG. The consistency of muscle activation trends between experimental data and model simulations was relatively high across the 4 exercises by eye inspection.

**FIGURE 7:**
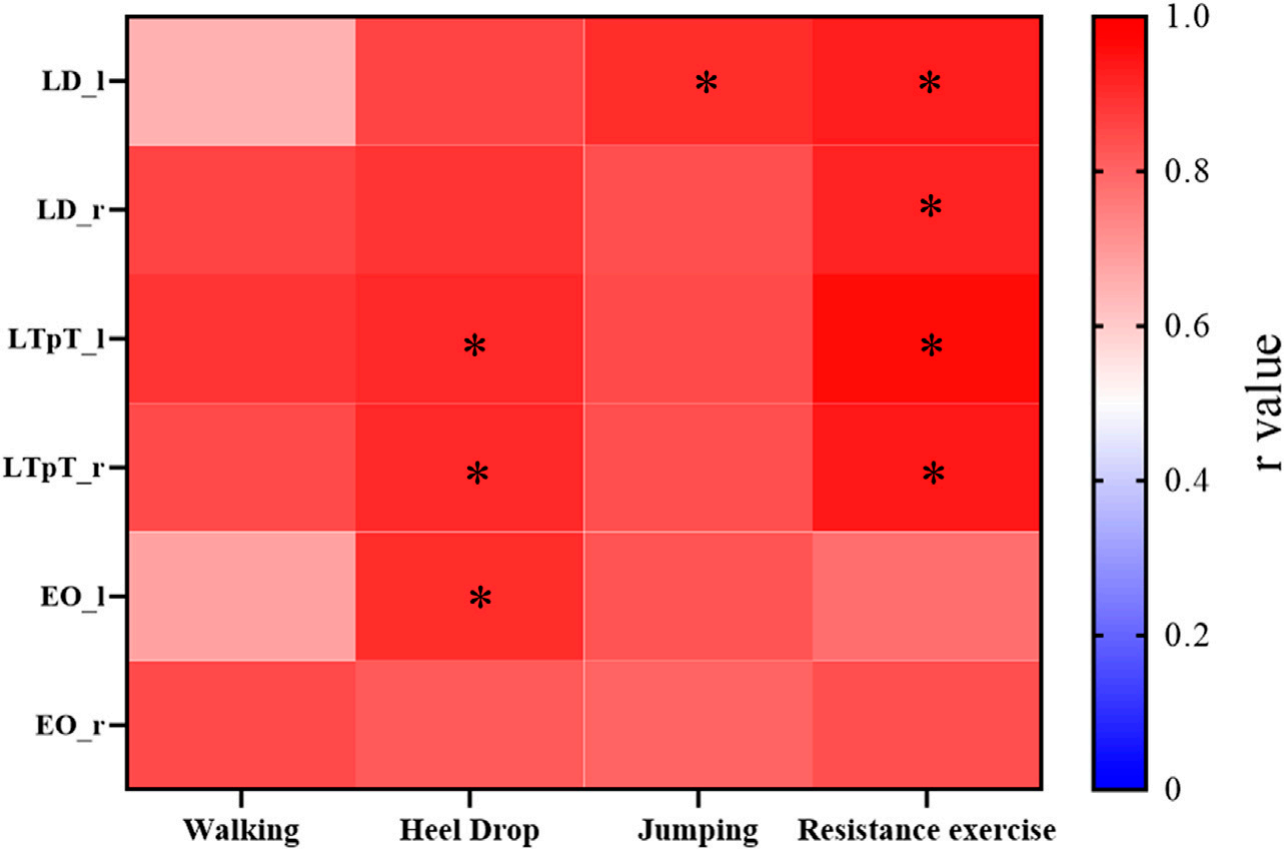
For the 4 types of exercises, the cross-correlation (r values) between the muscle activation obtained from the MS model and the average peak values of muscle activation processed from sEMG. *r > 0.9.

The correlations between the muscle activation obtained from the MS model and the mean peak sEMG muscle activation for 4 different exercises were also presented (Figure 7). Except for the left LD (r = 0.65) during walking exercise, the left EO (r = 0.68) during walking exercise, and the left EO (r = 0.78) during resistance exercise, the peak intercorrelation values for bilateral LD (LD_l, LD_r), LTpT (LTpT_l, LTpT_r), and EO (EO_l, EO_r) under different exercises were all above 0.80, reaching a maximum of 0.95.

### 3.3 Paravertebral muscle force

**FIGURE 8:**
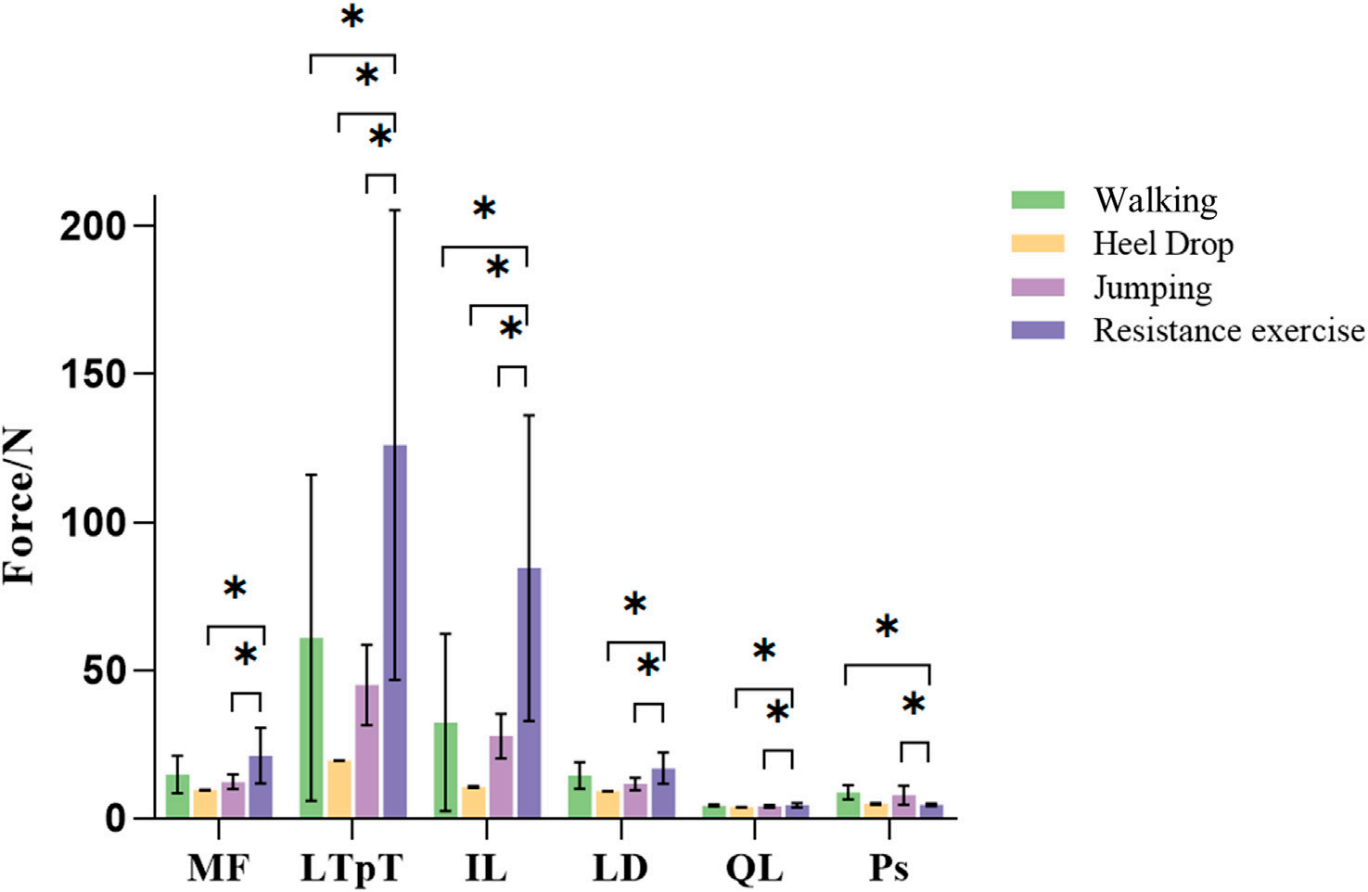
Different exercises of inferior paravertebral muscle force. MF: multifidus muscle; LTpT, longissimus thoracis; IL, musculi iliocostalis lumborum; LD, latissimus dorsi; QL, quadratus lumborum; Ps, psoas major.

MFs of six paraspinal muscles during the exercise were calculated using a semi-automatic in-house MATLAB algorithm (Figure 8). There were significant differences in the average paraspinal MFs during various exercises. Except for Ps, the average forces of the other 5 paraspinal muscles exhibited a pattern where resistance exercise yields the highest force with up to 126.04N, followed by walking exercise, and jumping exercise with the lowest force. During resistance exercise, the forces of the 5 paraspinal muscles were significantly greater than those during both heel drop and jumping exercises (*p* < 0.01), ranging from 1.22 to 8.18 times higher than heel drop and 1.17 to 3.11 times higher than jumping. Additionally, during resistance exercise, the forces of the LTpT and IL were significantly greater than the forces during walking exercise (LTpT: 128.42N vs. 60.97N, IL: 86.46N vs. 32.34N, *p* < 0.05). However, the force of the Ps during high and walking exercises were 7.82N and 8.82N, significantly greater than during resistance exercise, approximately 1.64 and 1.85 times higher (*p* < 0.05).

### 3.4 Intervertebral compressive force

**FIGURE 9:**
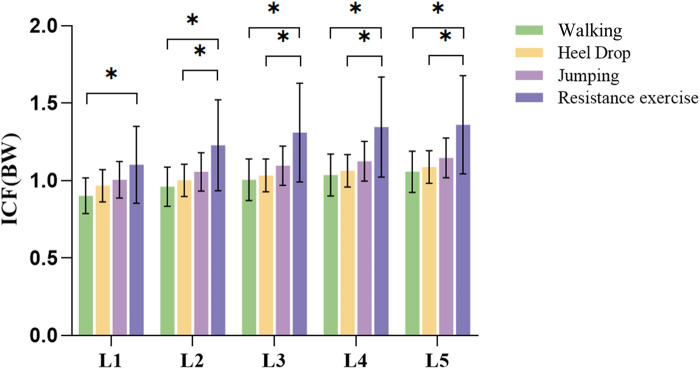
Average ICF (BW) of each lumbar segment during walking, heel drop and jumping and resistance exercise. *p < 0.05.

ICF for each segment during impact exercises and resistance exercise is shown in Figure 9. During resistance exercise, the ICF for the L1-L5 segments ranged from 1.1 ± 0.25 to 1.36 ± 0.32 BW, significantly greater than walking exercise (0.9 ± 0.12 to 1.06 ± 0.13 BW, *p* < 0.05). Furthermore, the ICF for the L2-L5 segments during resistance exercise was significantly greater than that of heel drop exercise (1.23 ± 0.29 to 1.36 ± 0.32 BW vs. 1.0 ± 0.12 to 1.09 ± 0.13 BW, *p* < 0.05). Additionally, peak ICF during the 4 exercises was analyzed, as detailed in Appendix Ⅰ. The results indicated that during jumping, the ICF for all vertebral segments was significantly greater than walking and heel drop exercise and resistance exercise (*p* < 0.05), approximately 1.50–1.91 times higher. Under heel drop exercise, all segmental peak ICF values were significantly greater than walking exercise (*p* < 0.05), approximately 1.23–1.27 times higher. Meanwhile, under resistance exercise, the peak ICF for the L3-L5 segments was significantly greater than walking exercise (*p* < 0.05), approximately 1.26–1.29 times higher. Spine ICFs were calculated based on the results from the SO (Table 2). The ICF exhibited a decreasing trend from L5 to L1 for all the exercises, but there were no statistically significant differences observed among the vertebral segments.

**TABLE 2:** Average intersegmental ICF (Mean ± SD, BW) of spine during walking, heel drop and jumping exercise and resistance exercise.

ICF (BW)	L1	L2	L3	L4	L5	Order
Walking	0.90 ± 0.12	0.96 ± 0.13	1.00 ± 0.13	1.04 ± 0.14	1.06 ± 0.13	L1 < L2 < L3 < L4 < L5
Heel drop	0.97 ± 0.10	1.00 ± 0.10	1.03 ± 0.11	1.06 ± 0.11	1.09 ± 0.11	
Jumping	1.00 ± 0.12	1.06 ± 0.12	1.10 ± 0.13	1.12 ± 0.13	1.15 ± 0.13	
Resistance	1.10 ± 0.25	1.23 ± 0.29	1.31 ± 0.32	1.35 ± 0.32	1.36 ± 0.32	

Abbreviations: Resistance represents resistance exercise (pulling elastic bands).

### 3.5 Correlations between spinal loads and ΔBMD

**FIGURE 10:**
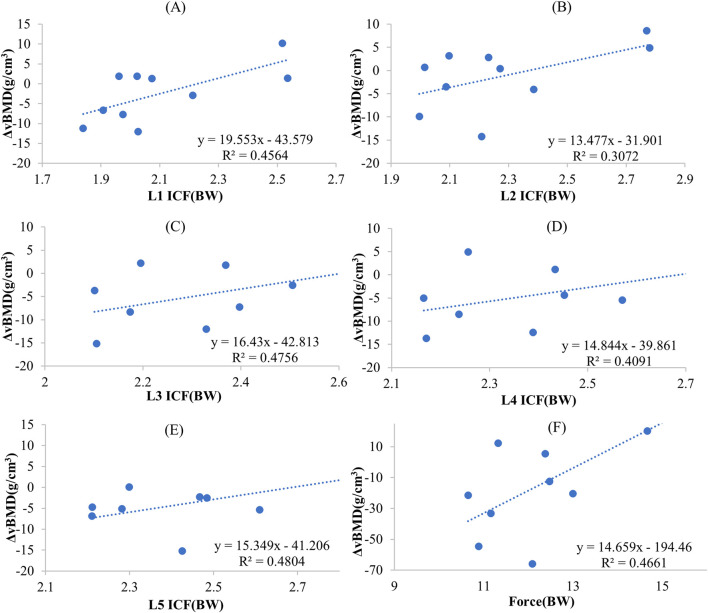
The regression-predicted ΔBMD curves for spine mechanical stimulation under different exercise paradigms are as follows: **(A)** Predicted ΔBMD curve for L1 ICF under combined exercise; **(B)** Predicted ΔBMD curve for L2 ICF under combined exercise; **(C)** Predicted ΔBMD curve for L3 ICF under combined exercise; **(D)** Predicted ΔBMD curve for L4 ICF under combined exercise; **(E)** Predicted ΔBMD curve for L5 ICF under combined exercise; **(F)** Predicted global spine ΔBMD curve based on the sum of paraspinal muscle force and ICF under combined exercise.

The correlation coefficients between the ICF caused by different exercises and ΔBMD for 5 segments were shown in Table 3. In the combined exercise ΔBMD was significantly correlated with ICF for all 5 segments. In the combined exercise, the correlation between the ICF of the L5 and L3 segments and ΔBMD was relatively high which was statistically significant (r = 0.693, *p* < 0.05; r = 0.690, *p* < 0.05). The L1 and L4 segments closely followed, with correlation coefficients of r = 0.676 and 0.640, respectively (*p* < 0.05). The correlation between the ICF of the L2 segment and ΔBMD was weaker and not statistically significant (r = 0.554, *p* = 0.096). Additionally, a correlation analysis was conducted between the change in ΔBMD of the global spine during combined exercise and the sum of MF and ICF of the paraspinal muscles adjacent to the L1-L5 vertebrae, revealing a significant positive correlation (r = 0.687, *p* < 0.05). The results of linear regression for the ICF of each spine segment and BMD during combined exercise are presented in Figures 10A–E and Figure 10F shows the BMD linear regression results, along with the sum of paravertebral MF and ICF of the spine.

**TABLE 3:** Correlation coefficients between ΔBMD and ICF of 5 spine segments.

Exercise	Correlation	L1	L2	L3	L4	L5
Walking	ICF and ΔBMD	0.698*	0.663*	0.458	0.528	0.401
Heel drop		0.381	0.476	0.382	0.469	0.445
Jumping		0.075	0.066	0.180	0.182	0.223
Resistance		0.623	0.509	0.596	0.550	0.586
Combined		0.676*	0.554	0.690*	0.640*	0.693*
Combined	ICF + MF and ΔBMD	0.687*				

Abbreviations: Resistance represents resistance exercise (pulling elastic bands); Combined represents combined exercise (jumping and pulling elastic bands); *p < 0.05. The bold values represent the coefficient of significant correlation between ICF and ΔBMD in combined exercise.

In the walking exercise, a moderate and significant correlation was observed between the ICF of the L1 and L2 segments and ΔBMD (r = 0.698, *p* < 0.05; r = 0.63, *p* < 0.05), while the correlation for the remaining segments was lower and not statistically significant. In the heel drop exercise, the correlation between the ICF of the L1-L5 segments and ΔBMD was weak (r = 0.381–0.476), and not statistically significant (*p* > 0.05). Similarly, during jumping exercise, the correlation between the ICF of the L1-L5 segments and ΔBMD was weak (r = 0.066–0.223, *p* > 0.05). In resistance exercise, a moderate correlation was observed between the ICF of the L1-L5 segments and ΔBMD (r = 0.509–0.623), but it was not statistically significant (*p* > 0.05).

## 4 Discussion

In this study, BMD changes was calculated after 6-month combined exercise intervention. Modified MS models were built for the first time to evaluate paravertebral MFs and ICF during combined exercise including jumping and resistance exercise and daily activities (walking and heel drop). The paravertebral MFs and ICF during jumping and resistance exercise were all significantly greater than daily activities (walking and heel drop) with up to 1.22–8.18 times. It was also found for the first time, the changes in spine BMD were significantly correlated with mechanical loads on the global spine and vertebrae generated by the combined exercise, compared with other activities.

The paired *t*-test results for lumbar spine BMD showed that after 6 months combined exercise intervention, the BMD of all lumbar segments decreased, but no statistically significant difference was found (*p* > 0.05). Previous studies have reported that the BMD of the lumbar spine in postmenopausal women who did not participate in exercise training would significantly decrease (Mean ± SD 0.007 ± 0.045 g/cm2, *p* = 0.002). Another research reported that each additional year after the final menstrual period was associated with 0.006 g/cm^2^ (*p* < 0.0001) lower LS (Shieh et al., 2022). All the above studies have shown that lumbar BMD spine in postmenopausal women will show a significant decline over time. Therefore, combined exercise intervention might slow down the loss of lumbar BMD. However, comparing with an increase in spine BMD after an 8-month high-intensity resistance and impact training (Kistler‐Fischbacher et al., 2021; Watson et al., 2015), a non-significant decline was observed. It might be due to the degradations of basal activity and/or functional response of osteoblasts, which were mediated by menopausal status and age-related alterations in postmenopausal women, may postponed their ability. The impairment of this ability contributed to a prolonged reversal phase in postmenopausal women with low BMD, which extended the opening time of the bone formation. Therefore, most of the newly formed bone with relatively low BMD might resulted in the underestimation of BMD changes.

Almost all paraspinal MFs during resistance exercises were greater than those during impact exercises. The 5 paraspinal MFs (MF, LTpT, IL, LD, QL) during resistance exercises were significantly greater than those during heel drop and jumping exercises, ranging from 1.22 to 8.18 times and 1.17 to 3.11 times. And the LTpT, IL forces during resistance exercise were significantly higher than in walking exercise. Therefore, it was proved that resistance exercise in this study can significantly activate the back muscle. The increase and activation of trunk muscles due to upper limb resistance exercise was also helpful in maintain an erect and correct posture (Oliveira and Gonçalves, 2009). Consequently, resistance exercise can increase the strength of the paraspinal muscle and generate greater paraspinal MF which affects the spine BMD (Bacelar et al., 2015). However, Ps force during walking and jumping were significantly higher than resistance exercise. The Ps is well known as a flexor of the hip which originates from the vertebral and inserts into the lesser trochanter of the femur (Bogduk et al., 1992; Yoshio et al., 2002). EMG studies show increasing activity with greater angles of hip flexion (Andersson et al., 1995). The hip flexion angle during walking and jumping exercises was higher than resistance exercise which may be the reason of Ps force during walking and jumping exercise significantly higher than resistance exercise.

ICF is another important biomechanical parameter during exercise. Previous research reported that the average ICF range at the L4 level during walking exercise was approximately 1.1 BW (Favier et al., 2021). This was consistent with our result of ICF at L4 calculated in this study (1.04 ± 0.14 BW), confirming the reliability of the MS model again. The ICF gradually increased from L1 to L5 during resistance exercise. A previous study reported that the ICF increased from L1 to L2, varied slightly from L3 to L4, and then reached the peak at L5 on bilateral load bearing (backpack) (Zhao et al., 2023). It has a similar trend with this study. In addition, the ICF also increased from L1 to L5, and the peak ICF during jumping exercise was significantly greater than walking, heel drop, and resistance exercise, ranging from 1.50 to 1.91 times (*p* < 0.05). This may be because impact exercise which introduced mechanical stimulation to the bones through the GRF (Favier et al., 2021), jumping exercise generated larger GRF and caused ICF transmitted to the spine from bottom to top.

After 6-month combined exercise, spine BMD remained at the same level with no significant decline observed, especially at L1, which is consistent with previous combined exercise interventions (Kistler‐Fischbacher et al., 2021; Hettchen et al., 2021). Notably, the paravertebral MFs and ICF calculated by the MS model for combined exercise were significantly correlated with ΔBMD of both global and individual segments, with correlation coefficients up to 0.693 (*p* < 0.05). However, no significant correlation in walking and heel drop exercises. It can be confirmed that, biomechanical factors such as MF and ICF was the main mechanical stimuli that cause spine BMD changes. However, regression analysis of ICF or the sum of ICF and MF with ΔBMD yielded R2 values less than 0.5, the fitting results of the regression analysis were unsatisfactory. This may be because the changes in spine BMD were influenced by other factors simultaneously. Thus, predicting BMD changes only based on paravertebral MF and ICF may not be accurate enough. Postmenopausal women’s spine BMD was also influenced by parathyroid hormones (Neer et al., 2001), conditions such as obesity, and metabolic syndrome, and higher serum ferritin levels can also reduce the risk of spine osteoporosis (Heidari et al., 2015). In addition, our study found a correlation between ICF in the L1 and L2 segments and ΔBMD during walking exercises (r = 0.698, *p* < 0.05; r = 0.663, *p* < 0.05), as shown in Table 2. L1 and L2 segments were the 2 segments with the lowest spine BMD, walking exercises may have a positive impact on these lower BMD segments. The MS model was able to analyze the exercise-induced mechanical loads, showing a potential in fast predicting exercise-induced bone and muscle changes. This method might apply to rehabilitation treatment to quickly analyze the rehabilitation effects of different exercises on the musculoskeletal system, finally optimize exercise programs.

The first limitation of this study was the number of participants. Due to the impact of the COVID-19 pandemic, the later stages of the exercise intervention were conducted online with remote supervision, resulting in poor compliance from some participants. With ten participants completed the intervention, it might have some effect on the validity of statistical inferences. Therefore, to take account of the effect of sample size, we tested the statistical power of *t*-test for BMD changes and Pearson correlations between BMD changes and kinetic data. High significant level was found for both *t*-test and correlation. Even with ten participants, this study showed a large effect size (F = 10.699, partial η^2^ = 0.121), which allows for good statistical power to detect significant differences. For the safety and ethical reason, participants with osteopenia were recruited to investigate the effect of exercise on BMD. Osteopenia was considered as precursor to osteoporosis with same symptoms which are low BMD and risk to fracture. The remodelling rate and metabolism were also the same with osteoporosis (Kenkre and Bassett, 2018; Martin and Seeman, 2008). Therefore, participants with osteopenia were able to fulfil the aim of our study to investigate the effect of exercise induced mechanical loading on BMD changes. The significant correlations result between exercise induced loading and BMD changes will not be affected. This pilot study provided a new insight to investigate the mechanism of exercise induced bone remodelling. We believe that this novel study of the effect of exercise induced loading on spine BMD changes provides the evidence needed to justify studying this question in a larger cohort. Secondly, the MS model was limited in obtaining the paravertebral MF on individual lumbar vertebrae. Therefore, a quantitative analysis of the relationship between the sum of ICF at individual vertebrae and paravertebral MF and the change in BMD was not performed. With the development of MS model, research might be able to investigate the relationship between individual vertebral joints and the sum of MFs with ΔBMD in the later studies. Finally, a non-significant change of BMD, rather than improvement was observed in this study. Besides, without a control group, the results of this study only can indirectly prove the positive effect of exercise. However, it was consistent with previous studies that exercise intervention improved the BMD of L1 (Vainionpää et al., 2005). It might be due to the time of designed intervention, but it has been found that bone remodelling cycle was approximately 4–6 months from study Zhao et al. (2017); Du et al. (2021b). In the future, a larger cohort study of longer exercise intervention should be designed to collect more clinical data regarding the effect of exercise on BMD changes.

## 5 Conclusion

In this study, modified MS models which were built for the first time to evaluate paravertebral MFs and ICF during high-impact and resistance exercise, which explained the mechanical stimuli of bone changes due to exercise. It was also found for the first time, compared to the daily activities, BMD changes at the spine were significantly correlated with spinal loading (MFs and ICF) caused by combined exercise. The use of the MS model provided a method to quickly estimate the 6-month exercise induced BMD changes, in the meantime, it shown a potential on predicting the mechanical stimuli of exercise induced bone remodelling. This study contributes to the understanding of exercise induced spine adaptation as well as a potential in fast prediction to evaluate the effect of physical exercise therapy.

## Data Availability

The raw data supporting the conclusions of this article will be made available by the authors, without undue reservation.
