# Supplementary material for: Musculoskeletal model predicted paraspinal loading may quick estimate the effect of exercise on spine BMD
Source: Front Bioeng Biotechnol. 2024 Dec 23;12:1464067. doi: 10.3389/fbioe.2024.1464067 (PMC11701238; doi:10.3389/fbioe.2024.1464067)

Supplementary Material

Appendix I: Peak ICF (BW) of lumbar vertebrae during 4 exercises. *p<0.05

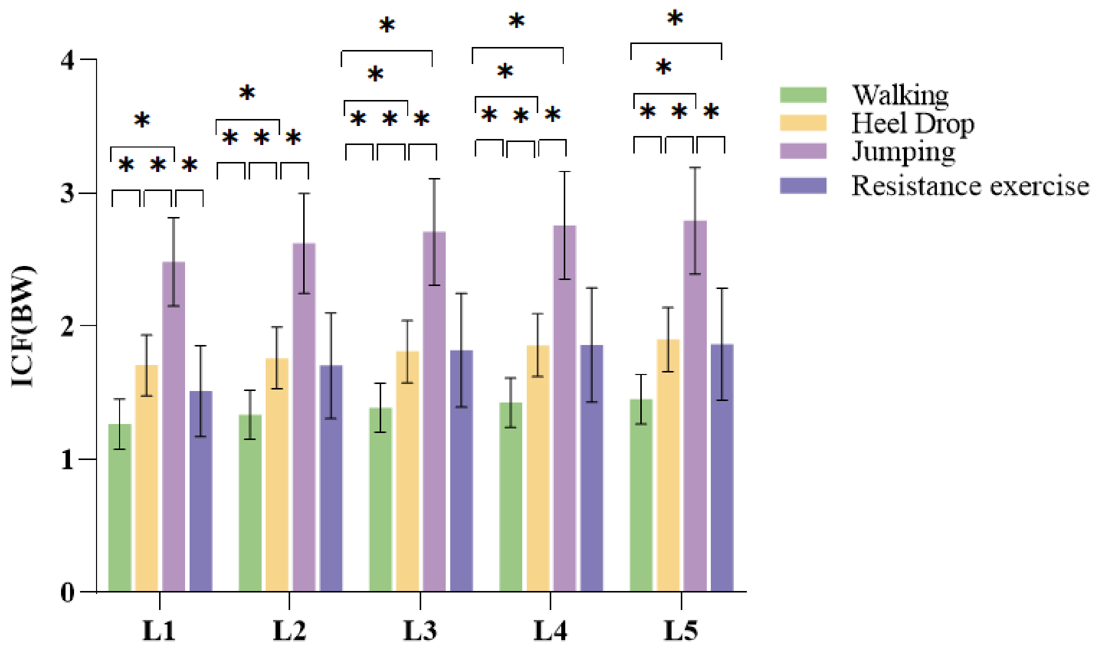

Appendix II: L1-L5 ICF curves during four different exercises

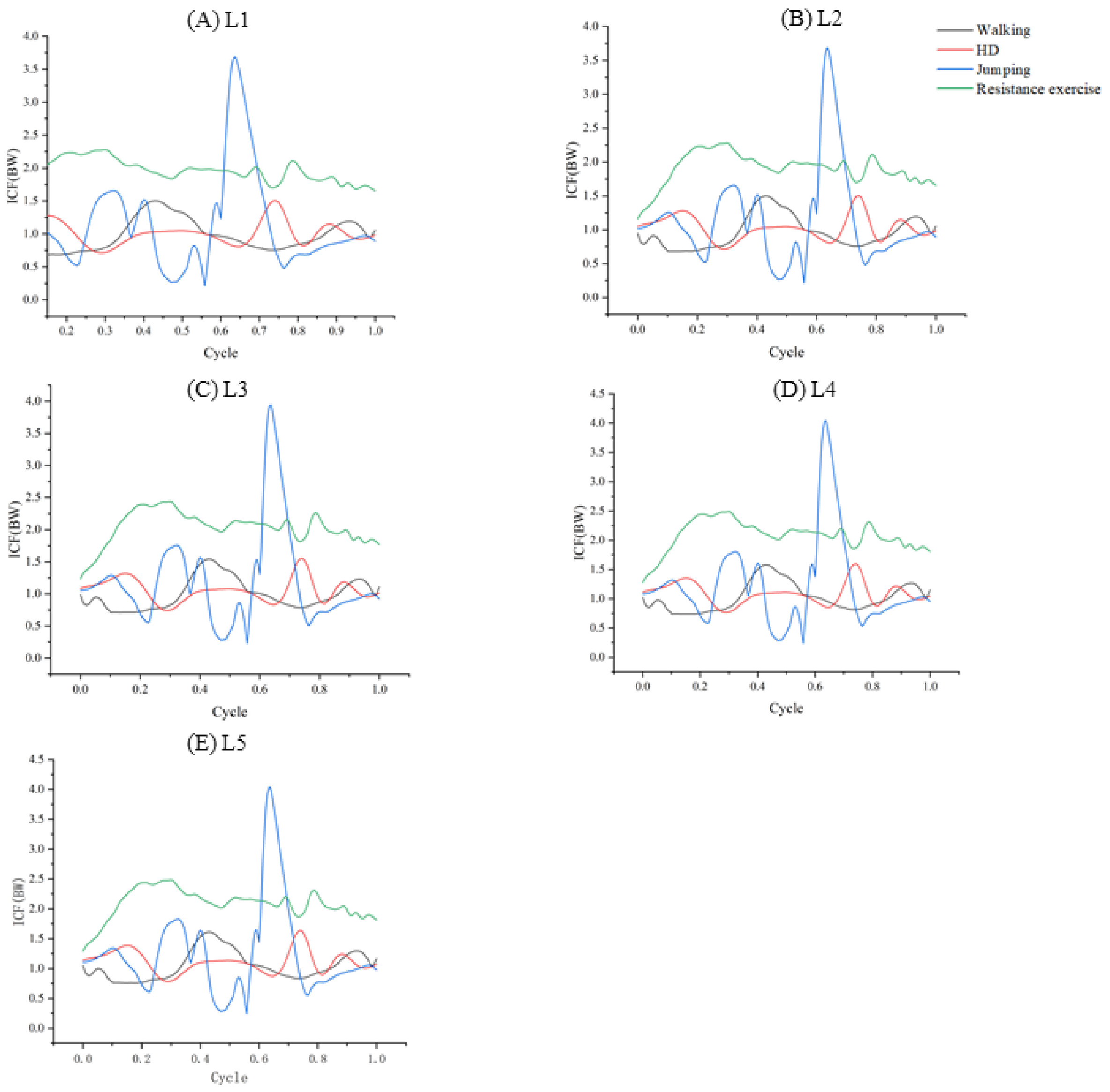

Supplement: Supplementary file 1 [file Image1.pdf]
